# Cardiovascular and Autonomic Responses after a Single Bout of Resistance Exercise in Men with Untreated Stage 2 Hypertension

**DOI:** 10.1155/2021/6687948

**Published:** 2021-03-29

**Authors:** Marcus Vinicius Machado, Thais de Paola Chequer Barbosa, Thais Camasmine Chrispino, Fabricia Junqueira das Neves, Gabriel Dias Rodrigues, Pedro Paulo da Silva Soares, Antonio Claudio Lucas da Nóbrega

**Affiliations:** ^1^Department of Biomedical Science, Ross University School of Veterinary Medicine, Basseterre, Saint Kitts and Nevis; ^2^Laboratory of Exercise Sciences, Department of Physiology and Pharmacology, Fluminense Federal University, Niteroi, State of Rio de Janeiro, Brazil; ^3^Department of Applied Nutrition, Federal University of Rio de Janeiro State, Rio de Janeiro, State of Rio de Janeiro, Brazil; ^4^Laboratory of Experimental and Applied Exercise Physiology, Department of Physiology and Pharmacology, Fluminense Federal University, Niteroi, State of Rio de Janeiro, Brazil

## Abstract

The aim of this paper is to assess the integrated responses of ambulatory blood pressure (BP), cardiac autonomic modulation, spontaneous baroreflex sensitivity (BRS), and vascular reactivity after a single bout of resistance exercise (RE) in men with stage 2 hypertension who have never been treated before. Ten hypertensive men were subjected to a RE session of three sets of 20 repetitions and an intensity of 40% of the 1-repetition maximum (RM) test in seven different exercises. For the control (CTR) session, the volunteers were positioned on the exercise machines but did not perform any exercise. Forearm blood flow was measured by venous occlusion plethysmography. We also analyzed the heart rate variability (HRV), ambulatory BP, blood pressure variability (BPV), and BRS. All measurements were performed at different timepoints: baseline, 20 min, 80 min, and 24 h after both RE and CTR sessions. There were no differences in ambulatory BP over the 24 h between the RE and CTR sessions. However, the area under the curve of diastolic BP decreased after the RE session. Heart rate (HR) and cardiac output increased for up to 80 and 20 min after RE, respectively. Similarly, forearm blood flow, conductance, and vascular reactivity increased 20 min after RE (*p* < 0.05). In contrast, HRV and BRS decreased immediately after exercise and remained lower for 20 min after RE. We conclude that a single bout of RE induced an increase in vascular reactivity and reduced the pressure load by attenuating AUC of DBP in hypertensive individuals who had never been treated with antihypertensive medications.

## 1. Introduction

Hypertension is one of the most prevalent chronic medical conditions in modern society, affecting more than one billion people in the world. Although hypertension often lacks symptoms, it is a major risk factor for the incidence of coronary artery disease, stroke, and all causes of mortality [1]. Several studies have also shown that, in essential hypertension, baroreflex sensitivity (BRS) is impaired, and it is associated with increased variability of ambulatory blood pressure (BP) and augmented response of BP to exercise [2].

Studies in humans, as well as in animal models, have demonstrated that, immediately after aerobic exercise, there are several changes in the mechanisms that regulate BP [3]. These changes result in postexercise hypotension (PEH) in both normotensive and hypertensive individuals [4]. While there is some evidence that resistance exercise (RE) also decreases resting BP, the mechanisms underlying this effect are not entirely understood. Previous studies have observed that the arterial baroreflex has been reset to defend blood pressure reduction after exercise. It contributes to increased sympathetic activity by decreasing the transmission of inhibitory signals to the pressure regulating center [5–7]. Nevertheless, despite the increase in sympathetic activity, a decrease in peripheral vascular resistance was observed, induced by the histamine receptor's activation, prolonged afferent muscle activation, and release of vasogenic agents such as nitric oxide or prostaglandins [8, 9]. However, since hypertension impairs central modulation and endothelial function, the consequences of RE on nontreated hypertension can likely be attenuated in these patients [10, 11].

Most people with different degrees of hypertension take prescribed medications to treat the disease, and autonomic nervous system, baroreflex mechanism, and vascular reactivity are potential therapeutic targets of several classes of antihypertensive drugs [12, 13]. Losartan, for example, improved BRS and heart rate variability (HRV) in essential hypertension after six months of treatment [13]. Furthermore, chronic cardioselective or nonselective *β*-blockade use in hypertensive patients increased BRS and decreased the pressor response to exercise [14].

A classic study carried out by Wilcox et al. [15] observed that hypertensive subjects using either atenolol (100 mg) or epanolol (200 mg or 400 mg) had an additive hypotensive effect induced by exercise compared to placebo individuals. A similar result was found with hypertensive women receiving captopril. In this study, a persistent reduction in BP was observed up to 10 h after RE, compared to a CTR session [16]. In another study, hypertensive individuals with intermittent claudication who were using different classes of antihypertensive drugs had a significant drop in systolic, diastolic, and mean BP after RE that lasted beyond 60 min [17].

Thus, the present study aimed to evaluate the integrated responses of ambulatory BP, cardiac autonomic modulation, BRS, and vascular reactivity after a single bout of RE in men with stage 2 hypertension who have never been treated with any antihypertensive drug before.

## 2. Materials and Methods

### 2.1. Ethical Approval

The Ethical Committee for Research at Antônio Pedro University Hospital, Fluminense Federal University, approved all procedures used in this study (CEP-CCM/HUAP 180/08). According to the Declaration of Helsinki, all individuals agreed to participate in the study through written informed consent obtained before participation.

### 2.2. Subjects

After an initial selection of 93 volunteers from our laboratory database, seventy-eight were excluded after a telephone interview. Five others were excluded after physical/blood examination because they did not meet the study's inclusion criteria. Ten hypertensive patients were then recruited for the study. The inclusion criteria for the study were the following: (1) age between 20 and 60 years, (2) stage 2 hypertension (systolic BP between 140 and 159 mmHg and/or diastolic BP between 90 and 99 mmHg) [18], (3) body mass index under 35 kg/m^2^, (4) being not physically active, (5) being free of medication (including all classes of antihypertensive), (6) normal resting electrocardiogram (ECG), and (7) absence of albumin in urine test.

Table 1 shows the anthropometric, metabolic, and hemodynamic characteristics of the subjects. Body mass index, fasting glucose, total cholesterol, and LDL-c were near-optimal values, but C-reactive protein was above normal values. Baseline BP values indicate stage 2 hypertension according to the Executive Summary of 2017 ACC/AHA/AAPA/ABC/ACPM/AGS/APhA/ASH/ASPC/NMA/PCNA Guideline for the Prevention, Detection, Evaluation, and Management of High Blood Pressure in Adults [18].

### 2.3. Experimental Procedure

**Figure 1:**
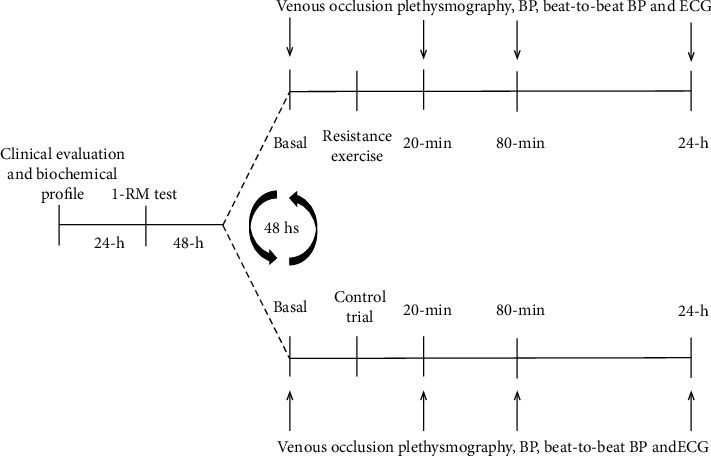
Schematic representation of the experimental protocol.

A complete description of the study design is presented in Figure 1. A preliminary clinical exam and biochemistry profile was performed after a 30 min supine resting period. Auscultatory BP was measured twice on both arms by an experienced measurer using a mercury column. Afterwards, they were submitted to a clinical exam (anamnesis, physical exam, 12 derivation ECG, and resting echocardiogram) and anthropometric evaluation (weight and height). A basic biochemical profile (fasting glucose, urea, C-reactive protein, triglycerides, total cholesterol, high-density lipoprotein (HDL), low-density lipoprotein (LDL), and very-low-density lipoprotein (VLDL)) was performed after a 12 h fast. Urine was collected to analyze albumin, abnormal elements, and sedimentoscopy.

After 24 hours of clinical exam and biochemical profile, the subjects performed 1-repetition maximum (1-RM) test for seven intercalating exercises for upper and lower limbs in the following order: chest press, leg extension, front pulldown, leg press, lateral raises, standing hip adduction, and standing knee flexion. There was a 30 min interval between exercises to allow the individuals to completely recover. The 1-RM tests were performed according to Kraemer and Fry's protocol [19]. Briefly, the maximum weight that can be lifted once was determined after no more than three attempts at each exercise. Subjects rested at least 3 min between each attempt. 48 h after 1-RM test, volunteers underwent RE and CTR in a randomized order at the same time in the afternoon, with 48 h intervals between the trials. The subjects refrained from caffeine, alcohol, and intense exercise for 24 h before the experiment. RE consisted of three sets of 20 repetitions with an intensity of 40% of 1-RM and 90 seconds of resting intervals between sets [20]. The 40% of 1-RM was chosen because a previous study showed low intensity (40–60% of 1-RM), and a large number of repetitions were more efficient in inducing PEH compared to high-intensity exercises [5]. In the CTR, volunteers were positioned on the exercise machines, but they did not perform any exercise. The instrumentation (≈30 min), session duration (≈1 h), and intervals were the same for both RE and CTR sessions.

At the end of both the RE and CTR, heart rate (HR) was recorded using a 12-lead electrocardiogram (ECG) (BioAmp, MLA2540, ADInstruments, Bella Vista, NSW, Australia). BP was obtained noninvasively using a servo-controlled finger plethysmography (Finapres Medical Systems, Netherlands). The model flow calculation was used to estimate cardiac output and derivate from the finger pressure pulse waveform (calibrated at the brachial artery) and assumed values for aortic diameter [21]. An oscillometric method using an automatized device (Dyna-MAPA, São Paulo, SP, Brazil) was also used to measure BP continuously for 30 min before (resting BP) and 24 h after both RE and CTR. The forearm blood flow (FBF) was measured by venous occlusion plethysmography (EC6, DE, Hokanson Inc., Bellevue, WA, USA). All assessments were made in the supine position at baseline, 20 min, 80 min, and 24 h after both experimental sessions.

### 2.4. Venous Occlusion Plethysmography

The FBF was determined by venous occlusion plethysmography [22] using calibrated mercury-in-silastic strain gauges, placed at the widest girth of the right forearm, and taken at baseline, 20 min, 80 min, and 24 h after each experiment. The right arm was supported in a comfortable position elevated approximately 5 cm above the level of the heart. One cuff was placed around the right wrist, and the other was placed around the right upper arm. The wrist cuff was inflated to 200 mmHg for one minute to isolate the vascular circulation of the hand, and it was kept inflated throughout the blood flow measurement [23]. The FBF was measured by rapidly inflating the cuff placed around the right upper arm to 50 mmHg, maintaining this pressure for 10 sec, and then rapidly deflating it to 0 mmHg for 10 sec, thus completing a 20 sec cycle. Six cycles of 20 sec were performed to determine the baseline FBF, totaling 120 sec for the baseline measurement. After the baseline FBF assessment, the cuff placed around the right upper arm was inflated to 200 mmHg for 5 min to occlude forearm circulation, thus provoking an ischemic stimulus. The wrist cuff was inflated to 200 mmHg once again at the fourth minute. At the end of the fifth minute, the arm cuff was deflated and a standard calibration pulse of 1 mV was generated, and, 10 sec after deflation, the forearm blood flow was measured for 3 min following the protocol previously described; that is, the upper arm cuff was inflated during 10 sec at 50 mmHg and deflated during 10 sec at 0 mmHg. Nine cycles were performed to determine forearm blood flow during reactive hyperemia.

Simultaneously, a cuff placed on the third finger of the left hand was used to measure noninvasive beat-to-beat BP continuously (Finometer, FMS, Netherlands). Forearm conductance and vascular resistance were calculated using blood flow and mean arterial pressure (MAP) and expressed as an arbitrary unit (a.u.), according to the following formula:

(1) $$\begin{array}{c} \text{Forearm Vascular Conductance}=\frac{\text{Blood flow}}{\text{MAP}},\\ \text{Forearm Vascular Resistance}=\frac{\text{MAP}}{\text{Blood flow}}\text{.} \end{array}$$

### 2.5. Spectral Analysis of Heart Rate and Blood Pressure

For the heart rate variability (HRV) and BP analysis, time series 300 events of pulse intervals and systolic BP were interpolated (cubic spline) and decimated to obtain a series equally spaced in time [24]. The series were submitted to a Fast Fourier Transformation and analyzed with specific software (Heart Scope II, AMPS LLC, Brescia, Italy). The frequency-domain indexes were the following: total power (TP, spectral density between 0.04 and 0.40 Hz), low-frequency power (LF, spectral density between 0.04 and 0.15 Hz), high-frequency power (HF, spectral density between 0.15 and 0.40 Hz), high-frequency normalized power (n.u.), and low-frequency normalized power (n.u.) [25]. Moreover, HRV time-domain indexes were analyzed by standard statistics by means of an algorithm developed in Matlab (Matlab 6.0, MathWorks Inc., USA) [26]: standard deviation of normal-to-normal (SDNN), the proportion of differences in successive NN intervals >50 ms (PNN50), and square root of the mean squared successive differences in pulse interval (RMSSD).

### 2.6. Spontaneous Baroreflex Sensitivity

Spontaneous baroreflex sensitivity (BRS) was evaluated by systolic BP (SBP) and R-R interval in time and frequency domains. The sequence method was used to evaluate spontaneous oscillations in SBP, and R-R sequences were characterized by a simultaneous increase or decrease of both. We used the SBP and R-R average sequences as a BRS index (BRS). In the frequency domain, the average of the square root sum of the R-R and SBP spectral power in the LF band was indicated as *α*-index (*α*-LF) [27].

### 2.7. Statistical Analysis

We computed the effective sample size and statistical power using the pilot study data. The statistical power of 80% was used to an alpha error of 0.05. Systolic blood pressure was used as the primary outcome (CTR day 144 ± 5 mmHg *versus* RE day 142 ± 5 mmHg, *n* = 4). A sample of at least six individuals was required to ensure a 95% confidence interval. Data distribution and homogeneity of variances were verified by Shapiro-Wilk and Levene's test, respectively. Logarithmic transformation was performed (when necessary) to allow the application of parametric tests. Two-way ANOVA, followed by the Student-Newman-Keuls post hoc test, was used between conditions (exercise day versus control day) and different moments (baseline, 20 min, 80 min, and 24 h). Data is presented as mean ± standard deviation. Statistical significance was considered when *p* < 0.05.

All analyses were performed using a Statistic program (version 8.0, StatSoft Inc., Tulsa, OK, USA, or GraphPad InStat 5.0, GraphPad Software, La Jolla, CA, USA).

## 3. Results

### 3.1. Ambulatory Blood Pressure

**Figure 2:**
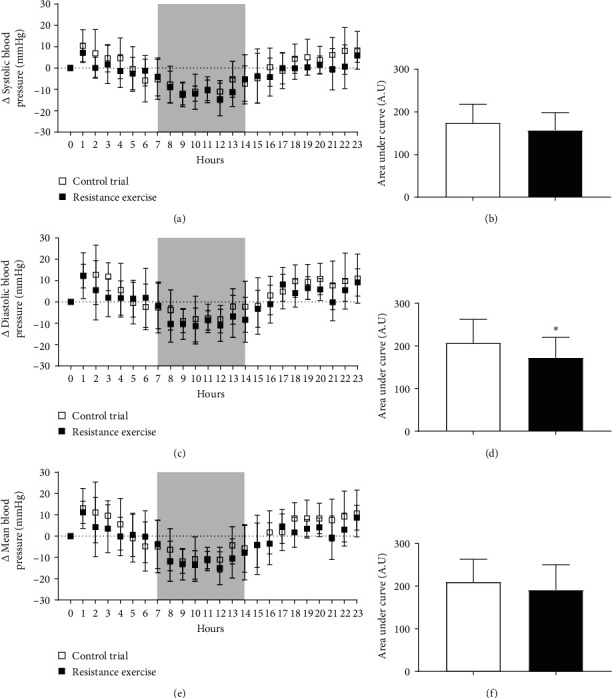
Systolic (A), diastolic (C), and mean BP (E) and respective area under the curve (B, D, and F) monitored 24 h after RE and CTR trial. The gray box represents sleep period.  ^*∗*^*p* < 0.05*versus* CTR trial.

Figure 2 shows BP behavior in both experimental sessions measured continuously every hour for 24 h. There were no differences in the delta of ambulatory blood pressure and sleep period of systolic, diastolic, and mean BP. Similarly, no differences were observed in the area under the curve of systolic and mean BP. Although the area under the curve of diastolic BP decreased 16.67% (*p* < 0.05) after the RE session, the effect size magnitude between the moments was considered small by Cohen's *d* test (*d* < 0.02).

Forearm Vascular Reactivity, Basal Forearm Blood Flow, and Forearm Vascular Conductance.

**Figure 3:**
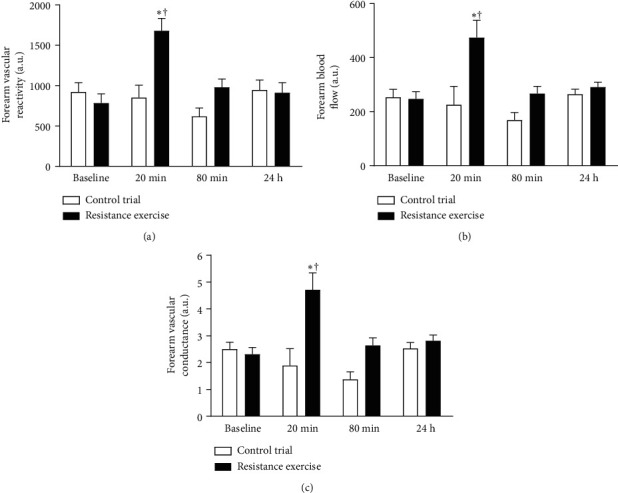
Forearm vascular reactivity (a), forearm blood flow (b), forearm vascular conductance (c) assessed by venous occlusion plethysmography before and after RE and CTR trial.  ^*∗*^*p* < 0.05*versus* baseline; †*p* < 0.05*versus* same moment of CTR trial.

Forearm vascular reactivity (FVR), forearm blood flow (FBF), and forearm vascular conductance (FVC) did not differ significantly from RE and CTR at baseline. However, FVR, FBF, and FVC were significantly increased up to 20 min after the end of RE (*p* < 0.05) (Figure 3).

### 3.2. Cardiac Autonomic Modulation and Baroreflex Sensitivity

HRV indexes are shown in Table 2. Despite low intensity of exercise, the HR increased up to 80 min after RE compared to the CTR session. HRV frequency (TP and LF) and time (SDNN, RMSSD, and pNN50) domains decreased up to 80 min after RE. No differences were observed in the HF (n.u) and LF (n.u) after RE, and there were no significant differences in BP variability indexes (data not shown).

**Figure 4:**
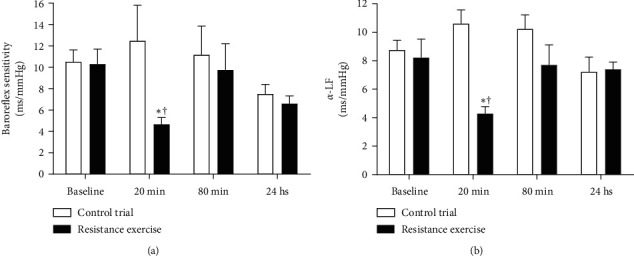
Baroreflex sensitivity index (a) and *α*-LF (b) assessed before and after RE and CTR trial.  ^*∗*^*p* < 0.05*versus* baseline; †*p* < 0.05*versus* same moment of CTR trial.

BRS was quantified by the HR response to changes in BP and determined by the variables *α*-LF and BRS index (Figure 4). RE reduced both variables (4.28 ± 1.58 and 5.72 ± 3.78 ms/mmHg) for up to 20 min after the end of the exercise compared to baseline (8.23 ± 3.85 and 11.24 ± 4.69 ms/mmHg; *p* < 0.05) and CTR trial (10.60 ± 3.04 and 13.49 ± 9.95 ms/mmHg; *p* < 0.05), respectively, for *α*-LF and BRS index, indicating BRS's impairment in the individuals evaluated.

## 4. Discussion

The present study investigated the effect of acute RE on autonomic and vascular responses in stage 2 hypertensive individuals who have never undergone treatment with antihypertensive medications. The main findings of this study are as follows: (i) PEH was observed only in diastolic BP after RE. (ii) The spectral analysis of HRV showed a reduction in the cardiac autonomic regulation of the heart (total power, HF, and LF) and attenuation of spontaneous BRS up to 80 min after RE. (iii) FVR, FBF, and FVC were significantly increased up to 20 min at the end of RE. (iv) The cardiac output increased up to 20 min after RE and it is probably involved in the attenuated PEH, even with the increase in the vascular reactivity.

### 4.1. Pharmacological Influence on Postexercise Hypotension

As demonstrated by our experimental data, although ambulatory BP did not decrease after exercise, we observed a reduction in the area under the diastolic BP curve, promoting a decrease in BP load to the arteries and heart. Despite this result's clinical relevance, our data do not corroborate previous studies that demonstrated a hypotensive effect of great magnitude after RE. For example, it was observed in a previous study that the association of low-intensity RE and captopril promoted a significant decrease in blood pressure up to 10 hours in hypertensive women [16]. Cavalcante et al. [28] verified a significant decrease in systolic and mean blood pressure up to 1 h after light (40% of 1-RM) and heavy (80% of 1-RM) load RE in an overweight woman with hypertension controlled by antihypertensive medication. Similarly, RE induced a greater PEH in hypertensive than in normotensive men. However, it is important to highlight that the hypertensive patients were submitted to two weeks of washout of antihypertensive medication, and, even with the outstanding PEH, this effect lasted for only 1 hour [29]. On the other hand, just a slight hypotensive response was observed after a single bout of RE in borderline hypertensive women that were not receiving antihypertensive medication [30].

Treatment of hypertension markedly reduces the cardiovascular risk and end-organ damage in prehypertension and stage 1 hypertension [31], but it is also known that some antihypertensive drugs can affect heart rate, sympathetic tone, maximal workload, and local lactate production during exercise [32, 33]. Furthermore, it has already been demonstrated that antihypertensive treatment improves not only BP but also functional sympatholysis in mild hypertension [34, 35]. Functional sympatholysis refers to a reduced vasoconstriction response to sympathetic activation in the contracting muscle [36]. This mechanism may improve muscle perfusion during exercise. However, there is substantial evidence that functional sympatholysis is impaired in both humans and animals with hypertension [34, 35]. In addition, some drugs can improve capillaries' structure and function and increase the number of capillaries in the skeletal muscle. It was recently shown that a new experimental drug, I_1_-imidazoline agonist, was able to reverse functional and structural capillary rarefaction in the skeletal muscle of obese rats, as well as reducing blood pressure, heart rate, and plasma catecholamine levels [37].

Thus, several studies support the hypothesis about the interaction between exercise and antihypertensive drugs, and it can be a plausible explanation for the different PEH magnitude and duration observed in previous studies [16, 29].

### 4.2. Vascular Reactivity, Heart Rate, and Cardiac Output

In this present study, vascular reactivity increased 20 min after RE even with an increased sympathetic drive. Previous studies have shown that sympathetic vasoconstriction is attenuated in exercising muscle by local metabolites and other substances that reduce vascular responsiveness to *α*-adrenergic receptor activation [36], but the PEH-related mechanism is mediated by reductions in both total peripheral resistance and sympathetic nerve activity [38].

Although RE improves vascular reactivity in hypertensive individuals, this type of exercise does not reduce the sympathetic influences on HR and cardiac output [39, 40]. HR remained higher 80 min after RE, while cardiac output remained elevated for approximately 20 min. Similar results were shown by several studies that indicate that HR remains elevated for few hours after RE, regardless of exercise intensity. This effect is mediated by the increase in sympathetic modulation and a decrease in parasympathetic modulation of the heart [5, 41]. However, this autonomic response may have directly influenced the magnitude of PEH. According to Halliwill [3], at the end of exercise, the cardiac output values are reduced more quickly than the vascular resistance recovers, causing an imbalance between these two determinants of BP, resulting in PEH.

### 4.3. Heart Rate Variability

The frequency-domain index of HRV (total power, LF, and HF) was reduced up to 20 min after RE. Total power reflects the overall autonomic activity, but changes in total power also influence LF and HF in the same direction, when these variables are expressed in absolute units [42].

Similarly, all time-domain variables (RMSSD, SDNN, and pNN50) were reduced up to 80 min during recovery after exercise. Time-domain variables reflect the parasympathetic branch function of the autonomic nervous system [1]. Thus, reduced time-domain values showed a vagal withdraw sympathetic after acute RE stimulation.

Previous studies have shown a large increase in sympathetic cardiac autonomic modulation up to 75 minutes after 40% ER and 80% 1-RM until fatigue. However, these results indicate that the increase in cardiac sympathetic modulation after ER depends on the exercise's intensity and the number of repetitions [5]. The high mechanical load on the vascular system in high-intensity exercises or that goes to fatigue increases the activation of mechanoreceptors, which in turn increases the activation of the metaboreflex by reducing blood flow [43].

### 4.4. Spontaneous Baroreflex Sensitivity

Baroreflex mechanism plays an essential role in the dynamic adjustment of circulation [44]. Our results demonstrate that BRS was reduced up to 20 min during exercise recovery in comparison with baseline measures. According to BRS, the vagal index of HRV (*α*-LF) decreased at the same moments of exercise recovery in comparison to baseline. In the spectral analyses, the total power and absolute values of high and low frequencies were lower in recovery than the baseline condition.

Previous studies reported similar hemodynamic and autonomic effects after RE in normotensive and hypertensive individuals [29]. Nonetheless, the authors investigated patients controlled by antihypertensive drugs. Thus, in the current study, the effect could be higher in essential hypertension without pharmacological treatment. On the other hand, a recent investigation did not show changes in the HRV index after RE in normotensive individuals, and, besides that, PEH did not occur in resistance modality [45]. This phenomenon was described only in aerobic exercise followed by resistance exercise.

## 5. Conclusion

In summary, we found that a single RE session induced an increase in vascular reactivity and reduced pressure load. Furthermore, apparently the increase in cardiac autonomic activity and attenuated spontaneous BRS after ER did not interfere with BP's dynamic or vascular reactivity. The results of the present study indicated that RE can reduce the pressure load by attenuating AUC of DBP in hypertensive patients who have never been treated with antihypertensive drugs.

From a practical point of view, even with the modest magnitude of changes in blood pressure observed in the study patients, a low-intensity ER routine for the whole body is recommended as a complement to treat hypertension at different stages of the disease. Moreover, based on the pool of different side effects observed by regular use of antihypertensive medications, RE looks like an efficient approach to treat the disease and lower medicine dosages.

### 5.1. Study Limitations

This study has limitations that must be considered. We did not have a group taking antihypertensive drugs for comparison with our experimental group to determine whether the drugs could positively affect PEH. Thus, future studies are required to clarify if the antihypertensive medication can impact the magnitude and duration of PEH after ER in hypertensive individuals.

## Figures and Tables

**Table 1:** Anthropometric, metabolic, and hemodynamic characteristics of subjects with untreated stage 2 hypertension (*n* = 10).

Variables			
Age (years)	43	±	9
Weight (kg)	88.6	±	17.1
Height (cm)	176.8	±	0.03
Body mass index (kg.m^−2^)	28.1	±	3.2
Glucose (mg.dL^−1^)	89.9	±	7.6
Urea (mg.dL^−1^)	32.4	±	6.1
Creatinine (mg.dL^−1^)	1.01	±	0.13
Cholesterol (mg.dL^−1^)	198	±	30
HDL (mg.dL^−1^)	48	±	15
LDL (mg.dL^−1^)	123	±	23
VLDL (mg.dL^−1^)	26	±	12
Triglycerides (mg.dL^−1^)	131	±	58
Microalbuminuria (mg.dL^−1^)	0.41	±	0.18
C-reactive protein (mg.dL^−1^)	0.45	±	0.88
SBP (mmHg)	144	±	7
MAP (mmHg)	120	±	5
DBP (mmHg)	96	±	4

Data presented as mean ± standard deviation; Abbreviations: HDL, high-density lipoprotein; LDL, low density lipoprotein; VLDL, very low-density lipoprotein; SBP, systolic blood pressure; MAP, mean arterial pressure; DBP, diastolic blood pressure.

**Table 2:** Spectral analysis of the heart rate variability before and after RE and CTR trial.

	Baseline	20 min	80 min	24 h
*Heart rate (bpm)*				
Control trial	62 ± 2	58 ± 2	58 ± 2	67 ± 1
Resistance exercise	64 ± 2	82 ± 2 ^*∗*^†	71 ± 1†	66 ± 1
*Total power (ms* ^*2*^)				
Control trial	3210.95 ± 2559.83	3943.32 ± 2312.90	4655.87 ± 2940.15	1885.68 ± 1095.88
Resistance exercise	3050.01 ± 2082.16	772.59 ± 765.27 ^*∗*^†	2044.91 ± 1305.78 ^*∗*^†	2042.24 ± 967.75
*LF (ms* ^*2*^)				
Control trial	1213.68 ± 910.16	1711.86 ± 1665.67	1968.06 ± 2397.95	863.02 ± 585.61
Resistance exercise	1240.59 ± 760.96	314.76 ± 367.01 ^*∗*^†	532.79 ± 248.99 ^*∗*^†	874.38 ± 429.65
*HF (ms* ^*2*^)				
Control trial	588.08 ± 428.68	635.88 ± 470.38	940.03 ± 711.08	312.36 ± 292.73
Resistance exercise	520.36 ± 332.92	147.19 ± 166.96 ^*∗*^†	547.73 ± 1004.91	349.30 ± 242.97
*LFn (n.u)*				
Control trial	69.31 ± 10.45	69.35 ± 10.65	63.36 ± 14.59	70.03 ± 16.43
Resistance exercise	65.90 ± 14.08	61.31 ± 17.05	61.66 ± 25.19	70.53 ± 10.69
*HFn (n.u)*				
Control trial	28.63 ± 10.00	28.88 ± 10.13	34.47 ± 14.47	25.91 ± 13.77
Resistance exercise	29.68 ± 11.38	31.14 ± 11.38	32.30 ± 18.91	26.15 ± 9.72
*SDNN (ms)*				
Control trial	62.9 ± 24	65.3 ± 35	74.5 ± 28	44.1 ± 29
Resistance exercise	61.9 ± 19	32.8 ± 24 ^*∗*^†	47.8 ± 16 ^*∗*^†	46.3 ± 23
*PNN50*				
Control trial	17.4 ± 5.7	21.8 ± 5.9	25.4 ± 8.7	7.5 ± 4.0
Resistance exercise	16.2 ± 5.8	3.6 ± 2.6 ^*∗*^†	9.5 ± 4.3 ^*∗*^†	10.1 ± 4.7
*RMSSD (ms)*				
Control trial	42.4 ± 18.4	46.8 ± 21.1	51.7 ± 19.7	28.0 ± 11.2
Resistance exercise	44.2 ± 19.0	19.3 ± 9.8 ^*∗*^†	31.3 ± 12.3 ^*∗*^†	30.6 ± 16.7

LF, low-frequency power; HF, high-frequency power; n.u. normalized units; SDNN, standard deviation of normal-to-normal; PNN50, proportion of differences in successive NN intervals >50 ms; RMSSD, square root of the mean squared successive differences in pulse interval.  ^*∗*^*p* < 0.05*versus* baseline; †*p* < 0.05*versus* same moment of CTR trial.

## Data Availability

The data used to support this study are available within this article.
